# Visual and auditory temporal integration in healthy younger and older adults

**DOI:** 10.1007/s00426-017-0912-4

**Published:** 2017-09-04

**Authors:** Jefta D. Saija, Deniz Başkent, Tjeerd C. Andringa, Elkan G. Akyürek

**Affiliations:** 1 Department of Otorhinolaryngology/Head and Neck Surgery, University Medical Center Groningen, University of Groningen, Groningen, The Netherlands; 2 Faculty of Mathematics and Natural Sciences, Artificial Intelligence and Cognitive Engineering (ALICE), University of Groningen, Groningen, The Netherlands; 3 Department of Psychology, Experimental Psychology, University of Groningen, Grote Kruisstraat 2/1, 9712 TS Groningen, The Netherlands; 4 Research School of Behavioral and Cognitive Neurosciences, University of Groningen, Groningen, The Netherlands

## Abstract

As people age, they tend to integrate successive visual stimuli over longer intervals than younger adults. It may be expected that temporal integration is affected similarly in other modalities, possibly due to general, age-related cognitive slowing of the brain. However, the previous literature does not provide convincing evidence that this is the case in audition. One hypothesis is that the primacy of time in audition attenuates the degree to which temporal integration in that modality extends over time as a function of age. We sought to settle this issue by comparing visual and auditory temporal integration in younger and older adults directly, achieved by minimizing task differences between modalities. Participants were presented with a visual or an auditory rapid serial presentation task, at 40–100 ms/item. In both tasks, two subsequent targets were to be identified. Critically, these could be perceptually integrated and reported by the participants as such, providing a direct measure of temporal integration. In both tasks, older participants integrated more than younger adults, especially when stimuli were presented across longer time intervals. This difference was more pronounced in vision and only marginally significant in audition. We conclude that temporal integration increases with age in both modalities, but that this change might be slightly less pronounced in audition.

## Introduction

Stimuli that rapidly succeed one after another can be perceived as a single composite stimulus and/or event. When watching a movie, for example, rapid, successive still images are perceived as fluent motion. This is due to a perceptual process named temporal integration, which combines stimuli within an interval up to about 200 ms into an aggregated representation (Hogben & Di Lollo, 1974; Di Lollo, 1980). The duration of the interval varies from person to person, however, and factors that affect cognitive functioning can play a role therein. A person’s age, then, can be an important factor, since aging results in an overall decline or slowing down of the cognitive system (Salthouse, 1996). Yet, how aging affects temporal integration specifically is not yet fully known.

In vision, several studies on temporal integration of visual forms have shown that older adults visually integrate across longer time intervals. For instance, Di Lollo, Arnett, and Kruk (1982) presented participants with two 5 × 5 dot matrices, presented simultaneously side by side, but with the successively plotted dots presented for just 1.5 μs. Participants were asked which of the two matrices contained a missing dot (Di Lollo et al., 1982). To find the missing dot, it is necessary to temporally integrate all dots as if they were presented simultaneously, because consolidating, let alone mentally comparing, 25 positions in such a short time would be impossible. The authors varied the total plotting interval by adjusting the interstimulus interval (ISI) between dots, and found that the younger group needed a shorter plotting interval (60.5 ms) to obtain the same level of 75% task performance as the older group (85 ms). This suggests that the older group temporally integrated the individual sequential dots over a longer interval than the younger group, indicating a longer temporal integration window.

Converging evidence has also been obtained with different tasks, such as color integration (fusion). Kline, Ikeda, and Schieber (1982) briefly presented participants with a green circle followed by a red circle, both presented in the same location. Perceptually, overlaying both circles would result in perceiving a yellow circle. By varying the ISI between the two circles, the authors could measure within what time window participants would temporally integrate the green and red circles and resultantly perceive a yellow circle. The authors found that the older group reported perceiving more color integrations up until the longest ISI, which amounted to a total stimulus duration of 90 ms. The younger group, in contrast, only reported seeing color integrations up to a total stimulus duration of 70 ms. Similarly, in a word recognition task, Kline and Orme-Rogers (1978) measured performance for three-letter words consisting of horizontal and vertical lines, by displaying two halves of random lines of each individual word sequentially. Recognizing the words becomes possible when a participant temporally integrates both halves in a single perceptual representation, which becomes easier when the ISI is small. Across a total stimulus duration range of 100–200 ms, the authors found that the older participants had higher word recognition scores with longer ISIs than the younger participants, which can be explained by a longer temporal integration window for the older group.

As alluded to, one explanation to why aging leads to increased visual temporal integration can be age-related cognitive slowing. According to the processing-speed theory, cognitive slowing would lead to carrying out fewer cognitive operations within a certain timeframe (Salthouse, 1996; Madden & Allen, 2015). When time is limited or processing time is externally constrained, later cognitive operations are then left with less processing time as earlier operations are taking longer to finish. In addition, due to cognitive slowing, memory traces of the results of earlier operations may decay before they can be used for later operations, which illustrates that cognitive slowing causes substantial ‘collateral damage’ apparent as noticeable impairments in daily life activities.

Given the fairly consistent results in the visual domain, one might expect that the auditory modality should be similarly affected. The supposed global nature of cognitive slowing is also compatible with that idea. To wit, measures reflecting other temporal aspects of vision and audition indeed change similarly with age: For both vision and audition, older adults have higher gap detection thresholds (Humes, Busey, Craig, & Kewley-Port, 2009) and are more susceptible to backward masking (Di Lollo et al., 1982; Gehr & Sommers, 1999). However, to our knowledge, there are no studies that have provided direct evidence that the auditory temporal integration window is longer for older adults. In fact, there is indirect evidence pointing to the contrary. An electroencephalographic study on the mismatch negativity (MMN; elicited by a violation in a to-be-expected order or identity of repetitive stimuli; Näätänen, Kujala, & Winkler, 2011) showed that the duration of the auditory temporal integration window does not differ between younger and older adults (Horváth, Czigler, Winkler, & Teder-Sälejärvi, 2007). Using two kinds of oddball experiments (double deviant and stimulus omission), the authors showed that the temporal integration window of their younger participants was around 250 ms, and the window of the older participants was around 200–250 ms.

The lack of evidence for prolonged auditory temporal integration leaves the possibility that aging might be affecting temporal integration differently for each sensory modality. The degree to which integration changes with aging might depend on the relative importance of time in a given sensory modality. In the visual modality, for instance, space is more dominant than time, and it is conceivable that the functionally weakest neurons (i.e., those dealing with temporal aspects) are the first to atrophy when people age. Analogous effects are seen in the body when age-related muscle atrophy is observed (Abate et al., 2007); the so-called “use it or lose it” principle (Schooler, 2007). In perception, the principal dimension of vision is space, but the principal dimension of audition is time (Kubovy, 1988; O’Callaghan, 2008). For example, the borders of visual objects are inherently indicated by coordinates in space, while those of auditory objects are defined in time. In addition, it is easier to imagine an object that is independent of time in the visual domain (e.g., a still image) than in the auditory domain. In line with these conjectures, Humes et al. (2009) showed that auditory gap detection thresholds are lower than the visual ones and that age differences appear to be larger for visual than for auditory stimuli.

Apart from a general effect of time, temporal integration might also be spared more specifically, because temporal integration is required on a daily basis to process and understand speech (Poeppel, 2003; Wallace & Blumstein, 2009): Especially, to analyze vowels, higher level processes map auditory information within 200 ms onto linguistic representations in the form of a phonetic category decision. In addition, even though research showed that older adults have more difficulties with understanding speeded speech (Wingfield, 1996; Gordon-Salant & Fitzgibbons, 2001), Schneider, Daneman, and Murphy (2005) showed that auditory decline and speed-induced stimulus degradation, but not cognitive slowing, may be responsible for lower intelligibility. Thus, it remains conceivable that age-related decline in temporal processing and integration might be lessened in the auditory domain.

### Current research

Taken together, there is substantial evidence, indicating that aging increases visual temporal integration, but for the auditory domain, the picture is less clear. Two possibilities exist: first, temporal integration may occur over longer intervals for the older population regardless of the specific sensory modality, which would seem compatible with the notion of general cognitive slowing. Second, differential aging effects on temporal integration in each modality may occur. Such a finding would suggest that the “use it or lose it” principle may apply, meaning that the visual modality could be affected by aging more than in the auditory modality, because the time dimension is less important in vision compared to the space dimension.

The main purpose of the present study was thus to investigate whether aging similarly affects temporal integration in both the visual and auditory domains. Clear evidence from a cross-modality comparison can only be provided with a task that provides a direct measure of temporal integration in each modality equally. In the present study, the visual and auditory tasks were made as similar as possible, using the rapid serial visual presentation (RSVP; Akyürek, Eshuis, Nieuwenstein, Saija, Başkent, & Hommel, 2012) task and its auditory equivalent, rapid serial auditory presentation (RSAP; Saija, Andringa, Başkent, & Akyürek, 2014a). For each task, we tested multiple stimulus durations (40, 70, and 100 ms). If aging affects temporal integration, then this should be reflected in older adults reporting more temporal integration for longer stimulus durations when two targets succeed each other directly (i.e., at Lag 1), in particular. More specifically, the number of temporal integration reports for older adults should decrease at a lower rate with longer stimulus durations compared to younger adults. This should then be reflected in a significant interaction effect of age and stimulus duration.

## Experiment 1A: Visual temporal integration

### Methods

#### Participants

**Fig. 1:**
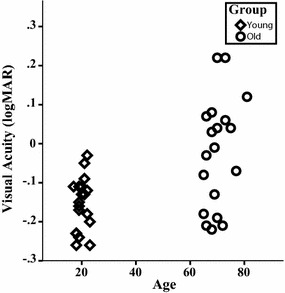
*Experiment 1A* This graph shows the visual acuity in LogMAR for young and older participants by age

Participants were naive to the purpose of the experiment. Since the experiment relied on visual stimuli, all participants were confirmed to have normal or near-normal vision according to the Ranges of Vision Loss established by the International Council of Ophthalmology (2002). The participants’ visual acuity was measured (with lenses or glasses if required) using the Landolt C test. The mean visual acuity for the young group was LogMAR −0.16 and for the older group LogMAR −0.02. Figure 1 shows the visual acuity as a function of age. Furthermore, mental flexibility and normal cognitive functioning were confirmed with the Trail-Making Test Parts A and B (Chanmugam, Triplett, & Kelen, 2013). Three older adults were excluded from participation, because one was suffering from macula pucker, one was unable to perform the task, and one had a stroke in the past. After exclusion, 19 young students of the University of Groningen (6 male and 13 female) with a mean age of 20 years (ranging from 17 to 23 years) and 19 older adults (16 male and 3 female) with a mean age of 70 years (from 65 to 81 years) participated in the study. Younger participants received course credit or monetary compensation, while older participants only received monetary compensation. Informed consent was obtained in writing before participation, and the study was approved by the Ethical Committee of the Department of Psychology at the University of Groningen.

#### Apparatus and stimuli

The experiment was implemented with E-Prime Professional 2.0.8.90 (Psychology Software Tools, Pittsburgh, PA) running on a desktop computer with Microsoft Windows XP. The visual stimuli were presented on a 19-inch CRT screen, which refreshed at 100 Hz with a resolution of 1024 × 768 pixels in 16-bit color, and which was placed at a viewing distance of approximately 60 cm. The participants’ responses were collected via a keyboard.

**Fig. 2:**
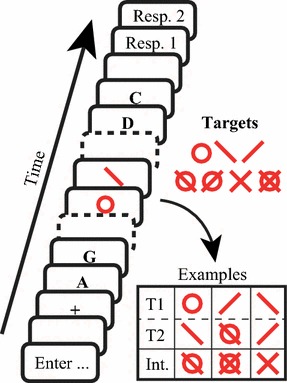
*Experiment 1A* Example of a typical trial to illustrate the procedure and visual stimuli. The *empty boxes* with *solid lines* represent blank periods of 100 ms. The *empty boxes* with *dashed lines* represent the succession of multiple distractor stimuli (i.e., *black letters*). The target stimuli were always presented in *red*. For each trial, all stimuli were of equal duration and were presented for 40, 70, or 100 ms. Each stimulus was separated by an ISI of 10 ms

The target stimuli consisted of the symbols **/ \ o** and their combinations, as shown in Fig. 2. They were at most 49 pixels in height and 33 pixels in width (approximately 1.6° and 1.1° of visual angle, respectively) and were displayed in red (RGB 255, 0, 0; 91 cd/m^2^). The targets were chosen, such that their features did not overlap with each other (e.g., **/** was never presented with the **X**). The distractor stimuli were drawn without replacement from the modern Latin alphabet (excluding I, J, K, L, O, and X to avoid confusion with the target symbols). The distractor stimuli, as well as the fixation cross, were all printed in bold 52 pt. Courier New font and colored in black (RGB 0, 0, 0; 2 cd/m^2^). The targets and distractors were about equal in size. The background color was always light gray (RGB 192, 192, 192; 265 cd/m^2^).

#### Procedure

The experiment consisted of a short block of practice trials and continued with 496 experimental trials with an optional break halfway, lasting for approximately 60–90 min. At 100 ms, after a trial was initiated by a participant, the fixation cross was displayed for 200 ms. Then, 19 stimuli succeeded each other, all of which were on screen for 40, 70, or 100 ms and followed by a 10 ms blank screen each (50, 80, and 110 ms SOA, respectively; 1/3 of trials each). On 94.4% of the trials, two of these stimuli were targets (T1 and T2), while the others were distractors. T1 appeared as either the fifth item or the seventh item in the stream and T2 followed T1 with either 0, 2, or 7 distractors in-between, referred to as Lag 1, 3, or 8 (31.5% of trials each). On 5.6% of the trials, T1 was a solo target.

The participants were told that each trial could contain one or two targets, and they were asked to identify each of them. After each stream, a 100 ms blank screen was presented, after which the participants were asked to enter the identity of T1 and then that of T2 on the numerical keypad. Each target response alternative was labeled on the numerical keypad. If a target was not spotted, then an empty response could be given by pressing the Enter key. However, guessing was encouraged when a participant was unsure about the identity of a target. Figure 2 shows an example of a trial that illustrates the procedure.

#### Analyses

Of main interest were the reports of integrated percepts (i.e., reporting the integrated percept of the combined features of T1 and T2) that were reported as a single response (i.e., no second response was entered). These responses were regarded as strict integrations, and indicated that the observer only perceived a single target, which constituted of the integrated combination of T1 and T2. Second, task performance was analyzed, which reflects correct response accuracy of the target identities and their temporal order. Analyses were performed on the number of trials in which T1 was correctly reported, and in which T2 was correctly reported given that T1 was correct as well (T2|T1). T1 was also considered correctly reported when the integrated percept of T1 and T2 was reported (as was T2|T1).

The data were in the form of count data, and because the variance of the data for each analysis was larger than the data’s mean, all data best fitted the negative binomial distribution. Therefore, the data were analyzed using generalized estimating equations using a negative binomial distribution with log link. For each analysis separately, the overdispersion parameter (*α*) was estimated and the working correlation matrix (WCM) was chosen based on the best goodness of fit [i.e., lowest quasi-likelihood under the independence model criterion (QIC); Pan, 2001]. Each analysis included the two within-subject variables’ stimulus duration (40, 70, and 100 ms) and T1–T2 lag (1, 3, and 8), as well as the between-subject variable age group (young and older participants). Strict integrations were expected to happen mostly at Lag 1 due to the short distance between targets and the lack of distractors in-between, and therefore, additional analyses were performed on the data of Lag 1 only, whereby T1–T2 lag was removed as a variable. For each test, a significance level of 0.05 was used.

The strict integration reports were represented as relative frequencies, that is, relative to all trials in which both target identities are retained regardless of their positions (i.e., strict integrations, order reversals, and both correct responses). For reference, the Appendix contains figures with the absolute integration rates for all experiments reported here. To account for this relativity, the offset for each combination of subject and condition was included in these analyses and was calculated as the natural log of the exposure (i.e., of the number of trials that include strict integrations, order reversals, and both correct responses, per subject and condition). For the (T2|T1) accuracy, the offset for each combination of subject and condition was calculated as the natural log of the exposure of the number of trials in which T1 was correct. For T1 accuracy, there was no relativity, so for each subject and condition, all trials could be included. Therefore, the T1 offset for all conditions and subjects was set to the natural log of the total number of trials per condition and subject [ln(52) ≈ 3.95].

The estimated marginal means of the analyses of relative frequencies of strict integration reports were plotted in bar graphs. The estimated marginal means of the T1 and (T2|T1) accuracies were also plotted in bar graphs, together with the accuracies when report order is ignored (e.g., when T1’s identity is correct regardless of T1’s position, namely, including T1 reported as T2, order reversals, and strict integrations).

### Results

**Fig. 3:**
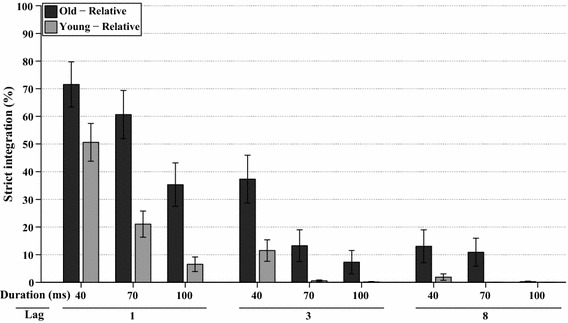
*Experiment 1A* This graph shows the estimated marginal means of the analyses of relative frequency of strict integrations for all combinations of stimulus duration, lag, and age group, as a percentage of the total number of trials in which both target identities were preserved. *Error bars* represent ±1 standard error of the mean

A full factorial analysis (WCM = autoregressive, *α* = 15.322) was performed on the relative frequencies of strict integration (i.e., relative to strict integrations, order reversals, and both correct responses), which are shown in Fig. 3. The frequency of strict integrations was significantly affected by lag, *χ*^2^(2, *N* = 342) = 64.7, *p* < 0.001, by stimulus duration, *χ*^2^(2, *N* = 342) = 95.5, *p* < 0.001, and by their interaction lag*duration, *χ*^2^(4, *N* = 342) = 55.5, *p* < 0.001. Figure 3 shows that reports of strict integrations are most prominent at Lag 1 and become less frequent with longer lags and longer stimulus durations. Strict integrations were also affected by group, *χ*^2^(1, *N* = 342) = 19.6, *p* < 0.001, as well as by the interactions of group*lag, *χ*^2^(2, *N* = 342) = 8.8, *p* < 0.015, and group*duration, *χ*^2^(2, *N* = 342) = 21.8, *p* < 0.001.

An additional analysis for Lag 1 only (WCM = unstructured, *α* = 3.029) showed that stimulus duration was a significant factor, *χ*^2^(2, *N* = 114) = 68.9, *p* < 0.001, which indicates that shorter stimulus durations resulted in more reports of strict integrations. Even more importantly, older adults were more influenced by stimulus duration than young adults, revealed by an interaction effect of group*duration, *χ*^2^(2, *N* = 114) = 26.7, *p* < 0.001, meaning that older adults integrated more often than young adults at longer stimulus durations. In addition, older adults reported more strict integrations at Lag 1 over all three durations, *χ*^2^(1, *N* = 114) = 17.6, *p* < 0.001. These effects can be seen in more detail, as shown in Fig. 3.

**Fig. 4:**
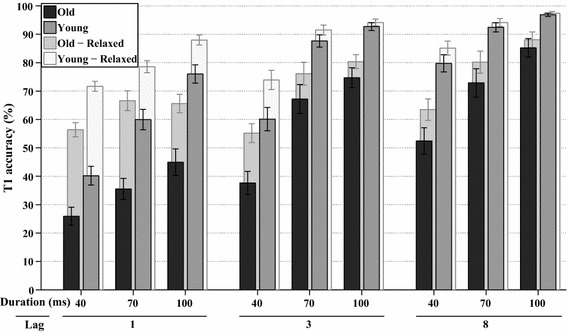
*Experiment 1A* The *solid bars* at the front show the estimated marginal means of the analyses on T1 task performance in percent correct, plotted for all combinations of stimulus duration and lag, for both age groups. The *transparent bars* at the back show the same analyses if report order is ignored (i.e., relaxed accuracy criterion). *Error bars* represent ±1 standard error of the mean

Another factorial analysis (WCM = unstructured, *α* = 2.015) was performed on the frequency of trials, where T1 was correct. The average accuracy of T1 per group for each lag and stimulus duration are shown in Fig. 4, together with the average accuracy when report order is ignored (i.e., relaxed criterion). T1 accuracy was significantly affected by lag, *χ*^2^(2, *N* = 342) = 213.3, *p* < 0.001, and stimulus duration, *χ*^2^(2, *N* = 342) = 137.8, *p* < 0.001, as well as by their interaction lag*duration, *χ*^2^(4, *N* = 342) = 43.3, *p* < 0.001. Figure 4 reveals that T1 accuracy was higher for each stimulus duration when lags were longer, as well as for each lag when the stimulus durations were longer. The accuracy of T1 also differed per age group, *χ*^2^(1, *N* = 342) = 21.4, *p* < 0.001, indicating that the younger group overall had higher performance. In addition, group*lag was significant, *χ*^2^(2, *N* = 342) = 8.6, *p* < 0.015, as well as group*lag*duration, *χ*^2^(4, *N* = 342) = 9.9, *p* < 0.045.

**Fig. 5:**
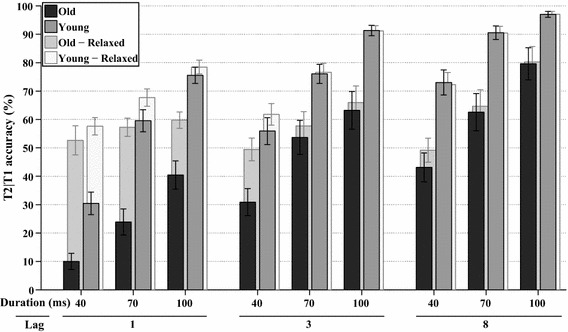
*Experiment 1A* The *solid bars* at the front show the estimated marginal means of the analyses on T2|T1 task performance in percent correct, plotted for all combinations of stimulus duration and lag, for both age groups. The *transparent bars* at the back show the same analyses if report order is ignored. *Error bars* represent ±1 standard error of the mean

A final full factorial analysis (WCM = independent, *α* = 2.667) was performed on the number of trials, where T2 was correct, given that T1 was correct as well (T2|T1). Figure 5 shows the average accuracy of T2|T1 per group and for each lag and stimulus duration, as well as the average accuracy when report order is ignored. T2|T1 accuracy was significantly affected by lag, *χ*^2^(2, *N* = 342) = 108.5, *p* < 0.001, and stimulus duration, *χ*^2^(2, *N* = 342) = 138.8, *p* < 0.001, as well as by their interaction lag*duration, *χ*^2^(4, *N* = 342) = 36.6, *p* < 0.001. Figure 5 reveals that T2|T1 accuracy was higher for each longer lag or longer stimulus duration. The accuracy of T2|T1 also differed per age group, *χ*^2^(1, *N* = 342) = 22.1, *p* < 0.001, indicating that the younger group overall performed better. In addition, group*lag was significant, *χ*^2^(2, *N* = 342) = 9.7, *p* < 0.01, as well as group*duration, *χ*^2^(2, *N* = 342) = 9, *p* < 0.015, and group*lag*duration, *χ*^2^(4, *N* = 342) = 14.7, *p* < 0.01.

Summarizing, older adults showed more integration than younger adults for visual stimuli, particularly for the longer stimulus durations tested. Elevated integration frequency was even observed at Lag 3, when 40 ms stimulus duration was used, for the older adults. For them, the speed of presentation seemed to overcome the inhibitory effects on integration of the intervening distractors. The younger group rarely integrated at Lag 3, even at the fastest presentation speeds. General task performance of the older adults, as measured by both T1 and T2|T1 accuracies, was also lower than that of the younger adults. Overall, the results were thus in line with expectations.

## Experiment 1B: The effect of retinal illuminance on visual temporal integration

To be able to interpret the results of Experiment 1A unambiguously, it is necessary to exclude the possibility that the observed age-related differences could be due to purely sensory factors, such as increasing opacity of the lens with age. Specifically, it is conceivable that older people integrates more, because their retinal illuminance is reduced (Coltheart, 1980; Di Lollo, Hogben, & Dixon, 1994). Older people have on average a reduction of around a 0.5 log unit of retinal illuminance compared to that of younger people (Weale, 1963). To investigate whether the older adults in Experiment 1A perceived more integrated stimuli because of an inverse intensity effect (i.e., more integration with dimmer stimuli), a new group of younger adults was tested with 34% screen brightness instead of 100% in Experiment 1B, which simulates an approximate 0.5 log unit reduction in retinal illuminance. The experiment was otherwise identical to Experiment 1A (young group only).

### Methods

#### Participants

**Fig. 6:**
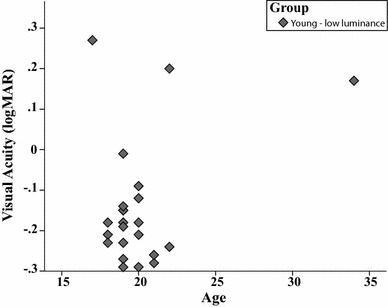
*Experiment 1B* This graph shows the visual acuity in LogMAR for the participants as a function of age

Twenty-three young students of the University of Groningen (20 male and 3 female) with a mean age of 20 years (from 17 to 34 years) participated. All participants had normal or near-normal vision: the mean visual acuity for this new group of young adults was LogMAR −0.14. Figure 6 shows visual acuity as a function of age. All participants received course credit for their participation.

#### Apparatus and stimuli

The only difference with Experiment 1A was that the brightness of the screen was set to 34% instead of 100% (in hardware), simulating reduced retinal illuminance as might be experienced by older observers. The red target stimuli were now displayed at 39 cd/m^2^ and the light gray background at 109 cd/m^2^.

#### Analyses

The analyses were focused on relative frequencies of strict integration reports. First, we tested whether the reduced brightness in Experiment 1B resulted in more strict integration reports than in Experiment 1A; therefore, the main analysis included the between-subject variable group (comparing the young participants from Experiment 1A with those from Experiment 1B) and the two within-subject variables’ stimulus duration (40, 70, and 100 ms) and T1–T2 lag (1, 3, and 8). Second, a detailed analysis was performed on Lag 1 with the within-subject variable stimulus duration, for both the young participants of Experiments 1A and 1B.

### Results

**Fig. 7:**
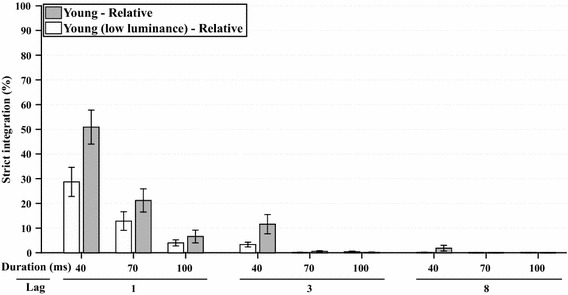
*Experiment 1B* This graph shows the estimated marginal means of the analyses of relative frequency of strict integrations for all combinations of stimulus duration, and lag, as a percentage of the total number of trials in which both target identities were preserved. The data from the young group of Experiment 1A (full luminance) are re-plotted next to the low luminance group for reference. *Error bars* represent ±1 standard error of the mean

A full factorial analysis (WCM = autoregressive, *α* = 24.360) was performed on the relative frequencies of strict integration, which are shown in Fig. 7. The frequency of strict integrations was significantly affected by lag, *χ*^2^(2, *N* = 378) = 100.387, *p* < 0.001, by stimulus duration, *χ*^2^(2, *N* = 378) = 30.09, *p* < 0.001, and by their interaction lag*duration, *χ*^2^(3, *N* = 378) = 19.41, *p* < 0.001. Figure 7 shows that reports of strict integrations were most prominent at Lag 1 and became less frequent with longer lags and longer stimulus durations, as observed previously. Strict integrations were also affected by the interaction of group*duration, *χ*^2^(2, *N* = 378) = 7.82, *p* < 0.025, and the interaction of group*lag*duration, *χ*^2^(2, *N* = 378) = 7.05, *p* < 0.035, reflecting that low luminance seemed to decrease integration frequency in some conditions only, particularly at Lag 1, and at 40 ms duration.

An additional analysis for Lag 1 only (WCM = unstructured, *α* = 31.216) showed that only stimulus duration was a significant factor here, *χ*^2^(2, *N* = 126) = 101.29, *p* < 0.001. The lack of a significant group factor indicates that luminance did not have a significant effect on strict integration reports.

Even though Experiment 1B could not perfectly match the retinal illuminance of older observers (e.g., due to constant room lighting), the reduction in screen luminance was substantial enough that a sensory-driven rise in integration should have been revealed. However, the findings did not at all support the idea that reduced retinal illuminance might have fostered integration in the current task. As shown in Fig. 7, there was actually a trend in the opposite direction: Reduced brightness resulted in the perception of fewer integrated stimuli. Therefore, we can conclude that older people do not temporally integrate more, because they perceive less brightness. The nature of the present task, in which dark stimuli appear on a light background (i.e., with inverse contrast), might have played a mediating role therein. In addition, it is conceivable that a reduced ability to perceive darker targets may actually have limited the opportunity to integrate, as integration requires at least the perception of the stimulus features.

## Experiment 2: auditory temporal integration

The auditory Experiment 2 was carried out after Experiment 1, its visual counterpart, produced the expected pattern of results. It was similar to the RSAP experiment described in Saija et al. (2014a) but with two additional stimulus durations (40 and 70 ms). Similar to the RSVP experiments, during the RSAP experiment, a participant was presented with a stream of auditory instead of visual targets and distractors. The participant then had to report which targets were heard. The two auditory targets consisted of complex tones, which could be integrated pairwise into two-formant synthetic vowels, analogous to the visual target combinations that were enabled in the RSVP experiments. During a pilot study with older participants, it became clear that they were unable to discriminate between the original target stimuli and remember them, maybe as a result of age-related changes in temporal fine structure processing (Füllgrabe, 2013), age-related short-term memory deficits (Chen & Naveh-Benjamin, 2012), or some loss of auditory acuity (even if within the range of normal hearing; Martini & Mazzoli, 1999). Therefore, the stimuli were modified in such a way that the older participants could discriminate the target stimuli more easily (as detailed below).

### Methods

#### Participants

**Fig. 8:**
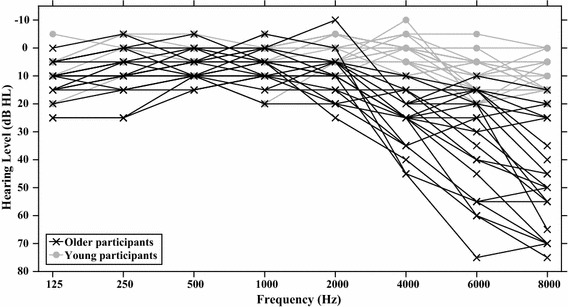
*Experiment 2* This graph shows the auditory acuity for the young and older participants in dB hearing level per frequency, plotted for the ear with the lowest hearing levels for each participant

Participants were naive to the purpose of the experiment. Since the experiment relied on auditory stimuli, all participants were selected to have normal or near-normal hearing. They reported to have normal hearing, and their audiometric thresholds were tested using the definition of normal hearing from Martini and Mazzoli (1999), namely, that the four-tone pure average across 0.5, 1, 2, and 4 kHz should be 20 dB HL or lower. Figure 8 shows the audiometric thresholds for each individual for both age groups. In addition, all participants were required to take the Trail-Making Test Parts A and B to test for mental flexibility and normal cognitive functioning (Chanmugam et al., 2013). An additional requirement was to be a fluent speaker of Dutch, as the stimuli were based on Dutch vowels. Two young and seven older participants were excluded from participation, because they found the training too difficult. In addition, eight older participants were excluded due to insufficient hearing, and two were excluded, because they were unable to successfully finish the Trail-Making Test Part B. After exclusion, 22 young students of the University of Groningen (11 male and 12 female) with a mean age of 20 (from 18 to 26) participated in the experiment for course credit. In addition, 22 older adults (7 male and 16 female) with a mean age of 65 (from 60 to 71) participated for monetary compensation. Informed consent was obtained in writing before participation, and the study was again approved beforehand by the Ethical Committee of the Department of Psychology at the University of Groningen.

#### Apparatus and stimuli

The experiment was implemented in Matlab (8.5.0.197613; R2015a) using Psychtoolbox (3.0.12; Brainard, 1997; Pelli, 1997; Kleiner et al., 2007) running on a Mac Pro with Mac OS X (10.10.4). Auditory stimuli were presented diotically through a Sennheiser HD 600 headphone, connected to an Echo Audiofire 4 external soundcard and a Lavry Engineering DA10 digital-to-analog converter. Responses were collected with a standard keyboard. Participants were tested in a sound-isolated booth.

**Fig. 9:**
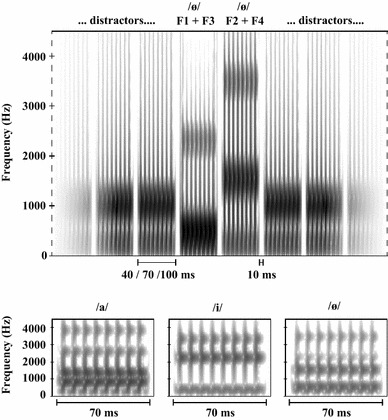
*Experiment 2* The top panel shows the spectrogram (window length = 5 ms; dynamic range = 70 dB) of a typical Lag 1 trial. From *left* to *right*, a number of distractor stimuli are presented, followed by part 1 (F1 + F3) of the vowel/ø/, and then part 2 (F2 + F4) of the same vowel, followed by more distractor stimuli. Stimuli were 40, 70, or 100 ms in duration, and were always separated by a 10 ms silent gap. The three *bottom panels* show spectrograms (window length = 5 ms; dynamic range = 45 dB) of the three 4-formant vowels/a/,/i/and/ø/, in this example with a duration of 70 ms

The stimuli were created in Praat using a Klattgrid (Weenink, 2009), which is a speech synthesizer based on the Klatt synthesizer (Klatt, 1980; Klatt & Klatt, 1990). The Klattgrid program was used to create three Dutch vowels/a/,/i/and/ø/ (Pols, Tromp, & Plomp, 1973) with a pitch tier of 120 Hz, as well as the distractor tone, which was always the same and repeated during the experiment. Each vowel consisted of the first four formants (F1–F4; see Table 1). The use of four formants instead of two as in Saija et al. (2014a) ensured that the artificial vowels sounded more rich and more similar to natural vowels, making them easier to recognize and to discriminate between them. Each artificial vowel was divided into two parts, and each part was a possible target sound. One part contained F1 and F3, and was perceived as being lower in timbre than the distractor, because most energy was at F1. The other part contained F2 and F4, and was perceived as being higher in timbre as most energy was at F2. F1 was lower in frequency than the distractor and F2 was higher (see Table 1). The bandwidth of F1 was set to 50 Hz, and the bandwidth of each subsequent formant was enlarged by 50 Hz compared to the previous formant. Part 1 was set at 65 dB SPL and each second part was set at a lower intensity (see Table 1) that would result in the best perception of the artificial vowel when both parts are combined. In addition, a ramp of 5 ms was placed at each on- and offset to prevent audible distortions of potential spectral splatter. The three bottom panels of Fig. 9 show spectrograms of the three vowels.

**Table 1:** Formant center frequencies and sound pressure levels of the formant combinations

	Distractor	/a/	/i/	/ø/
F1 center frequency (Hz)	1000	795	294	443
F2 center frequency (Hz)	–	1301	2208	1497
F3 center frequency (Hz)	–	2795	2294	2443
F4 center frequency (Hz)	–	3795	3294	3443
Part 1: F1 + F3 (dB SPL)	65	65	65	65
Part 2: F2 + F4 (dB SPL)	–	59	51	52

#### Procedure

The participants were asked to classify the targets as one of five response alternatives; the three different vowels, a tone that was lower in timbre than the distractor, or a tone that was higher than the distractor. All response alternatives were labeled on the numerical keyboard.

First, participants had to be trained to be able to identify all different targets. Therefore, they were given a few minutes to listen to each target (embedded in a short series of distractors) as often as they wanted until they felt acquainted with the targets. After that, they were given a short training session in which they were presented with the targets one by one. They then had to indicate which target they thought was presented, and they received visual feedback, together with an auditory presentation of the target they responded with and the presented target. Once the participants were able to distinguish the targets, a short final practice session followed consisting of a number of practice trials, which were similar to those in the experiment. Afterwards, the actual experiment started and consisted of 513 trials. A trial consisted of a series of 18 sequential stimuli, from which one or two could be targets and the rest distractors. On 94.74% of all trials, two targets were presented, in which both targets should belong to the same formant pair (i.e., T1 as F1 and T2 as F2, or vice versa). T1 appeared as the fifth or seventh stimulus, and T2 appeared at Lag 1, 3, or 8 (each 31.58% of all trials). On 5.26% of all trials, T1 was the single target, in which it could be a vowel (1.75%) or single formant (low tones 1.75%; high tones 1.75%). Stimuli had durations of 40, 70, or 100 ms (1/3 of all trials each), and were separated by a 10 ms gap. The top panel of Fig. 9 shows a spectrogram of a part of a typical Lag 1 trial.

The participants started a trial by pressing the spacebar. After each stream of stimuli, the participants entered what they heard as the first and second targets in their perceived order. When participants only heard a single target, they were able to give an empty response as the second target by pressing the Enter key. The experiment, including the training session, lasted approximately 1.5 h for the younger adults and 2 h for the older adults.

#### Data analysis

To classify a single response as a strict integration, the response should be the vowel that would have been the product of the combination of both targets. For example, if a participant reported to have only heard the/a/and no other target, and T1 was the F1 + F3 of/a/and T2 the F2 + F4 of/a/(or vice versa), then this report would be classified as a strict integration. Otherwise, the data analysis was similar to that of Experiment 1, except that the offset for T1 accuracy was ln(54) ≈ 3.99.

### Results

**Fig. 10:**
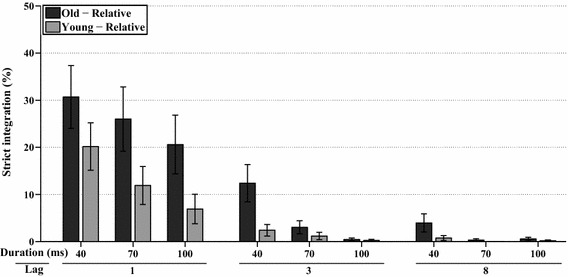
*Experiment 2* This graph shows the estimated marginal means of the analyses of relative frequency of strict integrations for all combinations of stimulus duration, lag, and age, as a percentage of the total number of trials in which both target identities were preserved. *Error bars* represent ±1 standard error of the mean

A full factorial analysis (WCM = exchangeable, *α* = 23.830) was performed on the relative frequencies of strict integration, which are shown in Fig. 10. The frequency of strict integrations was significantly affected by lag, *χ*^2^(2, *N* = 396) = 154.9, *p* < 0.001, by stimulus duration, *χ*^2^(2, *N* = 396) = 51, *p* < 0.001, and by their interaction lag*duration, *χ*^2^(4, *N* = 396) = 32.4, *p* < 0.001. As shown in Fig. 10, strict integrations were most frequent at Lag 1, and their frequency decreased with longer lags and longer stimulus durations. Strict integrations were also affected by group, *χ*^2^(1, *N* = 396) = 5.3, *p* < 0.025, as well as by the interaction of group*lag*duration, *χ*^2^(3 *N* = 396) = 9.3, *p* < 0.03.

An additional analysis for Lag 1 only (WCM = autoregressive, *α* = 2.56) showed that stimulus duration was a significant factor, *χ*^2^(2, *N* = 132) 15.3, *p* < 0.001, which indicates that shorter stimulus durations resulted in more reports of strict integrations. In addition, older adults marginally reported more strict integrations at Lag 1 over all three durations, *χ*^2^(1, *N* = 132) = 3, *p* = 0.085. These effects can be seen in more detail, as shown in Fig. 10.

**Fig. 11:**
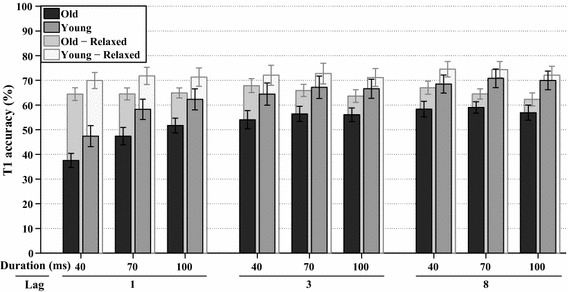
*Experiment 2* The *solid bars* at the front show the estimated marginal means of the analyses on T1 task performance in percent correct, plotted for all combinations of stimulus duration, lag, and age group. The *transparent bars* at the back show the same analyses if report order is ignored. *Error bars* represent ± 1 standard error of the mean

Another full factorial analysis (WCM = unstructured, *α* = 3.587) was performed on T1 accuracy, as shown in Fig. 11. T1 accuracy was significantly affected by lag, *χ*^2^(2, *N* = 396) = 74.2, *p* < 0.001, and stimulus duration, *χ*^2^(2, *N* = 396) = 34.5, *p* < 0.001, as well as by their interaction lag*duration, *χ*^2^(4, *N* = 396) = 86.5, *p* < 0.001. Figure 11 reveals that T1 accuracy was higher for each stimulus duration at longer lags, as well as for each lag when the stimulus durations were longer. The accuracy of T1 also differed per age group, *χ*^2^(1, *N* = 396) = 5.6, *p* < 0.02, indicating that the younger group performed better overall.

**Fig. 12:**
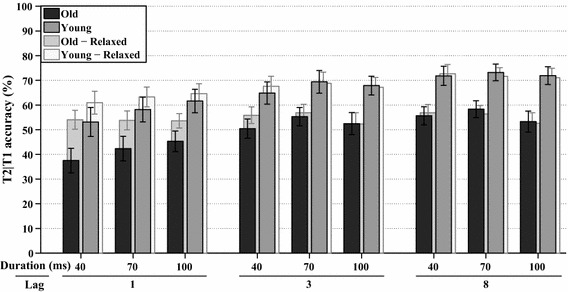
*Experiment 2* The *solid bars* at the front show the estimated marginal means of the analyses on T2|T1 task performance in percent correct, plotted for all combinations of stimulus duration, lag, and age group. The *transparent bars* at the back show the same analyses if report order is ignored. *Error bars* represent ±1 standard error of the mean

The last full factorial analysis (WCM = autoregressive, *α* = 3.757) was performed on T2|T1 accuracy, as shown in Fig. 1. T2|T1 accuracy was significantly affected by lag, *χ*^2^(2, *N* = 396) = 17.5, *p* < 0.001, and lag*duration, *χ*^2^(4, *N* = 396) = 13.6, *p* < 0.01. Figure 12 reveals that T2|T1 accuracy was higher for each stimulus duration when lags were longer, as well as for each lag when the stimulus durations were longer (except for Lag 3 and 8 from 70 to 100 ms). The accuracy of T2|T1 also differed per age group, *χ*^2^(1, *N* = 396) = 10.1, *p* < 0.002, indicating that the younger adults were also better able to identify the second target.

## Comparison of Experiment 1A and Experiment 2

To analyze whether temporal integration in both rapid serial presentation experiments occurred similarly, we performed a GEE test with experiment, age group, and stimulus duration as factors, on the strict integration data for Lag 1 only (WCM = unstructured; *α* = 6.807). The test revealed that experiment, *χ*^2^(1, *N* = 246) = 5.4, *p* < 0.025, age, *χ*^2^(1, *N* = 246) = 13.2, *p* < 0.001, and duration, *χ*^2^(2, *N* = 246) = 64.4, *p* < 0.001, was significant main factors, as expected. The significant interaction effects were experiment*duration *χ*^2^(2, *N* = 246) = 6.5, *p* < 0.04 and age*duration *χ*^2^(2, *N* = 246) = 16.7, *p* < 0.001. The significant effect of experiment indicates that temporal integration was more frequent in the visual domain, as is evident from comparing Figs. 3 and 10. The interaction effect of experiment and duration indicated that integration decreased more sharply as duration increased in the visual modality. The interaction between age and duration showed that overall, this decrease was attenuated for the older participants; they integrated comparatively more at the longer durations. However, the absence of interaction effects of experiment*age and experiment*age*duration indicates that age did not influence temporal integration differently per experiment. This means that aging affected temporal integration similarly in both modalities, even if it appeared from the individual analysis of Experiment 2 to do so less strongly in audition.

## General discussion

Previous literature provided evidence that aging results in more temporal integration in vision; however, evidence for the auditory domain remained inconclusive (e.g., Horváth et al., 2007). Therefore, the primary aim of this study was to investigate if aging affects temporal integration similarly in the visual and auditory domains. To this end, we conducted two rapid serial presentation experiments, visual and auditory, aiming to obtain a direct, comparable measure of temporal integration in each modality.

The results of the visual task (Experiments 1A and 1B) showed that temporal integration was significantly affected by aging at Lag 1. The older adults reported more temporal integration overall than the younger adults did. Most importantly, the interaction effect of age and stimulus duration at Lag 1 (where both targets succeeded each other directly) was significant. This showed that for older adults, visual temporal integration decreased less steeply with increasing stimulus duration, which means that the older adults integrated more at longer stimulus durations, as would be expected for a longer temporal window of integration. The results of the auditory experiment, however, showed a weaker aging effect on temporal integration: older adults reported only marginally more temporal integrations at Lag 1 than younger adults. In addition, there was no significant interaction effect between age and duration at Lag 1. Yet, the analysis of temporal integration at Lag 1 between both experiments revealed that the general pattern of performance was not reliably different. In other words, age influenced temporal integration similarly, even if temporal integration was most apparent for the visual modality as indicated by a significant main effect of experiment (cf. Figs. 3, 10). From these facts combined, we can conclude that aging does affect temporal integration in both the visual and auditory domains, but that the effect may be somewhat weaker in the latter.

### Locus of age-related differences in temporal integration

In the current experiments, we aimed to minimize the differences in visual and auditory sensory properties between the age groups, so that any differences in results could be attributed to differences in cognitive rather than perceptual capabilities (cf. Lindenberger & Baltes, 1994). Because it is not feasible to fully remove all sensory differences between the age groups, we aimed to reduce the differences to a minimum using participants that had normal vision and hearing according to the respective standards (International Council of Ophthalmology, 2002; Martini & Mazzoli, 1999). It must nonetheless be acknowledged that small sensory differences between the groups did remain, which might have contributed to differences in temporal integration. However, the results of Experiment 1B suggested that such a sensory effect can be discounted, since the data showed a pattern contrary to what would be expected if sensory degradation caused the age-related differences in temporal integration: we found less rather than more integration with reduced illuminance.

It, therefore, seems more likely that the differences in temporal integration originate from a more upstream locus in the perceptual processing pathway. For instance, older people have decreased early sensory memory abilities for short, individual stimuli (Fogerty, Humes, & Busey, 2016), making it harder to successfully keep fine-grained, individual stimuli in store. This might result in temporally blurred representations due to longer integration windows. Older people also seem to have more difficulties with separating and encoding short, individual, sequential stimuli because of decreased temporal processing capabilities, which might again result in overlapping representations. Supporting evidence has been obtained from gap detection tasks (Di Lollo et al., 1982; Humes et al., 2009), in which younger people can detect smaller gaps, and from temporal order judgments tasks, in which older people need longer ISI and stimulus durations to successfully judge the order of two sequential visual or auditory stimuli (Kolodziejczyk and Szelag, 2008; Ulbrich, Churan, Fink, & Wittmann, 2009).

Indeed, by themselves, such more function-specific theories are already quite capable of explaining why older people may have longer temporal windows and integrate more than younger people. However, it may be noted that the concept of cognitive slowing arguably encompasses these more specific theories. To recap, the processing-speed theory states that cognitive slowing leads to degraded cognitive functioning (Salthouse, 1996; Madden & Allen, 2015), which impacts perception according to the common cause principle. An individual with slower cognitive processing speed can carry out fewer cognitive operations within a certain timeframe (i.e., decreased temporal processing capabilities). Consequently, with limited processing time, subsequent cognitive operations are left with less processing time as earlier operations are taking longer to finish. Because of this, memory traces of the results of earlier operations may decay or become less strong, which make them susceptible for merging with subsequent memory traces. It, therefore, seems most parsimonious to refer more generally to cognitive slowing as the underlying mechanism that affects temporal integration with aging, regardless of the modality.

Although a general theory for the presently observed effects is appealing, the current data leave the possibility that the prominence of time in audition can at least slightly weaken the age-related differences in that modality. However, not all alternative explanations for this slight discrepancy between modalities can be ruled out. Because sensory and cognitive aging may correlate (e.g., Humes, Busey, Craig, and Kewley-Port, 2013; Roberts & Allen, 2016; Wayne & Johnsrude, 2015), the strict exclusion criteria applied out of necessity in Experiment 2 may have resulted in a relatively high-performing sample, which may have translated into comparatively modest integration rates. Thereby, the age-related effect may have become more difficult to detect. Another possibility is that the weaker effect in audition was due to the nature of the stimuli. One might suppose that the targets in the visual experiment were less meaningful than those in the auditory experiment (i.e., vowels) and that this difference could have mediated the integration process, such that auditory targets were less integrated. This account nevertheless seems problematic, because (1) not all auditory targets were meaningful vowels, (2) integrated reports could only consist of vowels combined from complex tones, which means that an increase in reports of integrated vowels should be expected, and (3) the symbols used in the visual experiment might also be regarded as meaningful (consider, for instance, the target “X”).

### Relation to neurophysiology and attentional blink

In neurophysiological terms, age-related cognitive decline is associated with myelin loss in the white matter of brain regions that myelinate late during brain development (Lu et al., 2011; Salami, Eriksson, Nilsson, & Nyberg, 2012; Lu et al., 2013), such as the prefrontal cortex (often associated with executive functioning, memory and attention) and the genu of the corpus callosum, which connects the prefrontal cortex on both hemispheres (Bloom & Hynd, 2005). Because the axons in these regions are less thickly myelinated, they are more fragile and sensitive to age-related degradation. In turn, such degradation diminishes the myelin’s function to accelerate transmission speed of action potentials through leaping conduction, which could possibly lead to cognitive slowing. Because the prefrontal cortex is related to attention and working memory, a general account of cognitive slowing thus fits our results quite well. Namely, in the currently used rapid serial presentation tasks, subjects need a sufficient level of attention and working memory capacity to successfully detect, identify, and remember the rapidly presented targets while ignoring intermediate distractors.

Furthermore, according to the simultaneous-type serial token model (Bowman & Wyble, 2007), two targets can be combined into a single target representation or episodic memory trace when the temporal overlap between the activation of both targets is adequate. Perceptually combining two targets in such a way costs less mental effort, as was shown by Wolff, Scholz, Akyürek, and van Rijn (2015), meaning that working memory is burdened less. Because older adults generally struggle more on attentional and cognitive tasks (Craik & Salthouse, 2011), it is conceivable that they use this temporal integration mechanism more frequently, as it may serve as a compensation mechanism to save mental resources. Most compensation mechanisms that are used by older adults result in increased brain activity compared to younger adults, to compensate for the age-related changes in the brain (Grady, 2012). In our tasks, to successfully detect, identify, remember, and keep up with the rapidly presented targets and ignore distractors, it is conceivable that older adults integrate more, because they have less mental resources or neuronal connections to perform this demanding task.

If so, it might be hypothesized that the brain activity of older adults in the prefrontal cortex increases as a way to keep up with the fast pace, resulting in an attempt to increase attention to the targets. Previous research showed that if more attention is given to targets temporal integration also increases (Visser & Enns, 2001), and also that successful temporal integration is related to increased amplitudes of the N1, N2, and late P3, which are event-related potential components related to attention (Akyürek, Schubö, & Hommel, 2010). Note that even though temporal integration might come with increased brain activity, it is nonetheless less demanding (or costs less mental effort) than keeping up with each single stimulus at a time, making it a suitable compensation mechanism for older adults with fewer neuronal connections (and thereby likely fewer mental resources) to begin with. In practice, such compensation would result in a prolonged temporal integration window, as longer periods are covered in a single episodic memory trace, which can be seen in our experiments, where the older adults integrated more at longer durations.

These interpretations fit well with the previous attentional blink (AB) results. The AB is expressed in the difficulty of perceiving the second of two targets (typically in RSVP) if it arrives between 150 and 500 ms after the first (Raymond, Shapiro, & Arnell, 1992). Importantly, recent work on individual differences suggests that people with a larger AB tend to integrate more (Willems, Saija, Akyürek, & Martens, 2016), which is in line with task performance in terms of effective allocation of cognitive resources, as given above. Furthermore, the previous research has also shown that the AB is larger for older adults in both modalities (Lahar, Isaak, & McArthur, 2001; Slawinski & Goddard, 2001). The current results show a similar pattern, both for integration, as discussed, and for target accuracy also: age had a significant effect on T1 and T2|T1 accuracies in both modalities, meaning that for both measures, older adults had lower accuracy over all conditions. One caveat is that even though we controlled for normal visual and auditory acuity, in practice, the acuity was on average slightly better for the younger groups, which might have contributed to the differences in accuracy.

Finally, a further advantage of a prolonged temporal integration window, besides the reduction of mental effort, is that it might be beneficial for high-level compensatory mechanisms for better perception of degraded speech, such as measured in studies of the phonemic restoration effect (Warren, 1970; Başkent, 2012). With phonemic restoration, listeners are able to restore degraded speech that contains missing speech segments that are filled by loud noise, using top–down knowledge to fill in the missing segments and combine the available and filled-in loose segments into coherent understandable speech. Saija, Akyürek, Andringa, and Başkent (2014b) showed that older adults, in some conditions, have a larger restoration effect than younger adults, and concluded that this might be due to the older adults’ superior language skills, vocabulary, and world knowledge. However, in light of the current results, it might be that temporal integration plays a role as well. Namely, Fig. 10 shows that with auditory stimuli, older adults integrated more at Lag 3 than younger adults (most prominent at 40 ms stimulus duration, and similar to the visual task). Normally, temporal integration would occur when two targets are presented in succession without intermediate distractors. However, for the older adults in this case, integration also happened with intermediate distractors at Lag 3. Such integration of two targets spanning over two intermediate distractors is not seen with young adults. With phonemic restoration, listeners also have to combine information of speech segments that are separated or masked by intermediate noise. Therefore, it is conceivable that a prolonged temporal integration window, as is seen with older adults, might have a positive effect on the phonemic restoration ability.

## Conclusion

In summary, the current results show that the older adults integrated overall more than the young adults, independent of modality. The older adults also integrated comparatively more at longer durations than the young adults. This effect was most clearly observed in the visual domain, and seemed less pronounced in audition. These results seem to reflect a general, cognitive–perceptual change with age, with the tentative addition that the prominence of time in audition may weaken this effect for auditory temporal integration.

## Figures and Tables

**Fig. 13:**
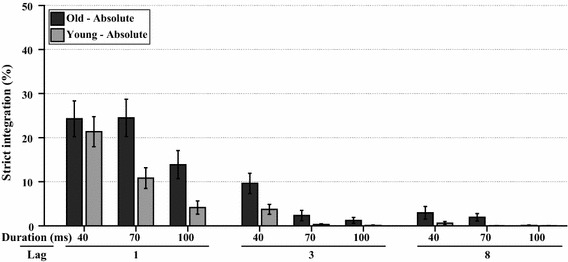
*Experiment 1A* Estimated marginal means of the analyses of absolute frequency of strict visual integrations for all combinations of stimulus duration, lag, and age group, as a percentage of the total number of trials in which both target identities were preserved. *Error bars* represent ±1 standard error of the mean

**Fig. 14:**
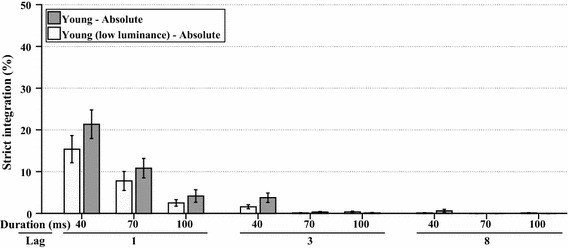
*Experiment 1B* Estimated marginal means of the analyses of absolute frequency of strict visual integrations for all combinations of stimulus duration and lag, as a percentage of the total number of trials in which both target identities were preserved. The data from the young group of Experiment 1A (full luminance) are re-plotted next to the low luminance group for reference. *Error bars* represent ±1 standard error of the mean

**Fig. 15:**
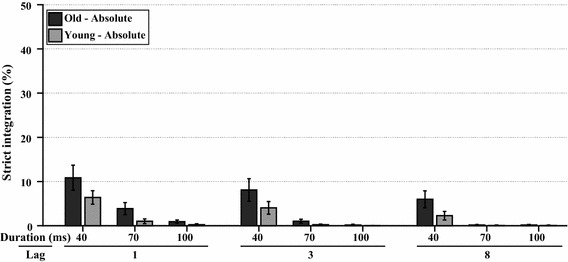
*Experiment 2* Estimated marginal means of the analyses of absolute frequency of strict auditory integrations for all combinations of stimulus duration, lag, and age group, as a percentage of the total number of trials in which both target identities were preserved. *Error bars* represent ±1 standard error of the mean

## Appendix

See Figs. 13, 14, and 15.
